# Organotypic three-dimensional assays based on human leiomyoma–derived matrices

**DOI:** 10.1098/rstb.2016.0482

**Published:** 2017-11-20

**Authors:** Tuula Salo, Mauricio Rocha Dourado, Elias Sundquist, Ehsanul Hoque Apu, Ilkka Alahuhta, Katja Tuomainen, Jenni Vasara, Ahmed Al-Samadi

**Affiliations:** 1 Cancer and Translational Medicine Research Unit, University of Oulu, Oulu 90014, Finland; 2 Medical Research Centre, Oulu University Hospital, Oulu, Finland; 3 Department of Oral and Maxillofacial Diseases, University of Helsinki, Helsinki 0014, Finland; 4 Helsinki University Hospital, Helsinki 0014, Finland; 5 Department of Oral Diagnosis, Oral Pathology Division, Piracicaba Dental School, University of Campinas, Campinas 13414-903, Brazil

**Keywords:** *in vitro* cancer invasion, 3D, drug testing

## Abstract

Alongside cancer cells, tumours exhibit a complex stroma containing a repertoire of cells, matrix molecules and soluble factors that actively crosstalk between each other. Recognition of this multifaceted concept of the tumour microenvironment (TME) calls for authentic TME mimetics to study cancer *in vitro*. Traditionally, tumourigenesis has been investigated in non-human, three-dimensional rat type I collagen containing organotypic discs or by means of mouse sarcoma-derived gel, such as Matrigel^®^. However, the molecular compositions of these simplified assays do not properly simulate human TME. Here, we review the main properties and benefits of using human leiomyoma discs and their matrix Myogel for *in vitro* assays. Myoma discs are practical for investigating the invasion of cancer cells, as are cocultures of cancer and stromal cells in a stiff, hypoxic TME mimetic. Myoma discs contain soluble factors and matrix molecules commonly present in neoplastic stroma. In Transwell, IncuCyte, spheroid and sandwich assays, cancer cells move faster and form larger colonies in Myogel than in Matrigel^®^. Additionally, Myogel can replace Matrigel^®^ in hanging-drop and tube-formation assays. Myogel also suits three-dimensional drug testing and extracellular vesicle interactions. To conclude, we describe the application of our myoma-derived matrices in 3D *in vitro* cancer assays.

This article is part of the discussion meeting issue ‘Extracellular vesicles and the tumour microenvironment’.

## 1. Introduction

The migration and invasion of cancer cells into adjacent tissue are important initial steps in carcinogenesis, which can lead to metastasis formation at secondary sites [1]. Solid tumours are generally considered invasive once they begin to penetrate the surrounding extracellular matrix (ECM) and multiple layers of mesenchyme [2]. The transformed epithelial cells may invade via the epithelial–mesenchymal transition, where cancer cells gain multiple attributes enabling invasion and metastases. Cancer cell invasion may arise individually (elongated-mesenchymal, rounded amoeboid and spike-mediated) or collectively (multicellular streaming, tumour budding and collective invasion) [3,4].

The surrounding neoplastic stroma of cancer cells—that is, the tumour microenvironment (TME)—is an active player in the multistep process of the invasion–metastasis cascade [3]. The interaction between cancer cells and TME leads to several changes in the structure and protein content of solid tumours. All TME compounds—including cells, ECM, soluble factors and extracellular vesicles (EVs)—collectively with cancer cells are crucial in affecting the complex processes of cancer invasion and metastases.

In experimental cell biology, cell migration and invasion represent distinct phenomena. Migration is the directed movement of cells that do not pass through obstructive barriers, whereas invasion necessitates the destruction of barriers in order to pass through them and thus by necessity is accompanied by ECM remodelling [5].

Here, we first briefly summarize the main carcinoma TME components including cancer-associated fibroblasts (CAFs), immune cells, blood vessels, matrix molecules, proteases and EVs. Second, we describe the properties of our human uterus leiomyoma tissue–derived matrices, myoma discs and Myogel. Finally, we provide examples of their application in three-dimensional (3D) cancer migration and invasion assays as well as in cancer drug testing.

### (a) Cancer-associated fibroblasts

CAFs are found in the TME of most solid tumours. While these cells were recognized long ago, their origin is not yet definitively known. Several theories were proposed and several types of cells were suggested as the origin of CAFs, including resident fibroblasts, mesenchymal stem cells and malignant epithelial cells [6–9]. Some suggested that CAFs represent the leading cells in tumour invasion allowing cancer cells to follow [10]. As such, CAFs play a primary role in tumour development, growth and metastasis [11]. Furthermore, their presence predicts poor survival in mobile tongue carcinoma, especially when located around tumour islands [12,13]. However, CAFs are not always related to a poor prognosis, at least not in pancreatic cancers [14].

Several markers can be used to identify CAFs, such as α-smooth muscle actin (SMA), fibroblast-specific protein 1, fibroblast activation protein (FAP), desmin, platelet-derived growth factor β (PDGF-β) receptor and fibroblast growth factor (FGF) [15,16]. Some of these CAF-related markers, such as αSMA, FAP and the PDGF-β receptor, have been associated with poor prognosis for colorectal and pancreatic cancers [15]. In addition, CAFs are known to support cancer metastasis [17]. Interestingly, CAFs are also found in the lymph nodes in secondary tumours associated with oral carcinomas. Yet it remains unclear whether those CAFs accompany cancer cells or if they are differentiated *in situ* at the lymph node [12].

### (b) Immune cells

Large amounts of inflammatory cells are located within a tumour, which are closely related to tumour progression or suppression [18]. Inflammation within the tumour can be divided into acute and chronic conditions, where, in general, the former is known to decrease cancer risk and the latter increases it [19]. Recent advances in tumour immunity could clearly distinguish two types of tumour-infiltrating immune cells: the tumour-associated myeloid cells, mainly representing innate immunity, and those usually working in favour of tumour progression [20,21]. On the other side, in patients with high T cells, representing adaptive immunity, infiltrated tumours show better prognosis [22,23]. Interestingly, in oral tongue cancer the high infiltration of CD163+ Foxp3+ CD80+ cells altogether was associated with high recurrence rate [24]. Inflammation participates in every stage of tumour development by secreting cytokines, chemokines, growth factors, prostaglandins and reactive oxygen and nitrogen species. Tumour progression partly depends on the balance between anti-tumorigenic and pro-tumorigenic immune and inflammatory factors. As a result of their importance in TME, immune cells were used to develop a new strategy for cancer treatment called immunotherapy [25].

Cancer immunotherapy research is rapidly growing and attracting wide interest. Based on boosting the patient's immune system to eliminate cancer cells, immunotherapy surpasses conventional chemotherapy in its specificity and improves therapy-related morbidities. The mechanism of immunotherapy depends on the immune system, which ranges from boosting the entire immune system to directing specific immune cells towards the tumour. Several immunotherapies are already in the clinical-trial stage and some are already used clinically. Between these immunotherapies, immune checkpoint inhibitors against CTL4, PD-1 and PD-L1 were found to be the most successful candidates [26]. Advances in immunotherapy research have already greatly improved the management of many cancer types. Cancer responses to immunotherapies remain inconsistent, from highly effective in some patients to completely ineffective in others [27,28]. Thus, we need to better understand the interaction between cancer and immune cells as a part of TME, whereby additional *in vitro* and *in vivo* studies are needed.

### (c) Angiogenesis

Tumour progression requires a constant supply of nutrition and oxygen. Therefore, in theory, neo-angiogenesis—the formation of new capillaries—is an essential process for tumour growth and metastasis [29]. Angiogenesis depends on a balance between promoters and inhibitors [30]. TME serves as a storage space for factors that affect angiogenesis. Among these, vascular endothelial growth factors play a key role in tumour angiogenesis [31]. In turn, VEGF affects several functions, including inducing angiogenesis, stimulating proliferation, acting as a survival factor and preventing endothelial cell apoptosis, regulating vascular permeability, promoting chronic inflammation and healing wounds [31,32].

Tumour blood vessels differ from physiological angiogenesis, having more loosely attached pericytes that express desmin and α-SMA [33]. Tumour neovascularisation remains heterogeneous and includes capillary sprouting, convoluted and excessive vessel branching, distorted and enlarged vessels, erratic blood flow, microhaemorrhaging, leakiness and abnormal levels of endothelial cell proliferation, and apoptosis [3]. These features of tumour blood vessels explain why tumours are often hypoxic despite being highly vascularized [34]. In addition to VEGF, also several other factors are suggested to induce tumour angiogenesis, such as transforming growth factor (TGF)-α, FGF-3 and hepatocyte growth factor (HGF) [35].

### (d) Matrix molecules and proteases

The composition of the TME matrix greatly affects the physical, biochemical and biomechanical properties regulating cancer cell behaviour. The TME matrix composition differs between cancers, but is primarily composed of structural proteins and soluble factors. Structural molecules include various collagens, fibronectin, laminins, proteoglycans, hyaluronic acid and tenascin. The soluble components include, for example, cytokines, growth factors and cryptic molecules liberated from the TME matrix [36]. Many of these components are synthesized by fibroblasts or released by proteases secreted by CAFs or cancer cells. Indeed, tumourigenesis overturns ECM, liberating many molecules that support cancer cell invasion and proliferation.

Matrix metalloproteases (MMPs) consist of a family of 24 endopeptidases that are overexpressed in several tumours. MMPs are the main modifiers of TME because they can hydrolyze most of the primary matrix macromolecules [37,38]. Most MMPs are pro-angiogenic, pro-invasive and pro-metastatic due to their ability to modulate ECM, thus allowing cancer cells to penetrate into the surrounding tissue. MMPs also liberate chemokines and growth factors from ECM affecting cancer progression. However, some, such as MMP-8, also play a protective role because they can liberate anti-tumorigenic molecules inhibiting the spread of cancer [39]. MMPs are regulated via the activation and inhibition of the tissue inhibitors of metalloproteinases (TIMPs). The net proteolytic activity of MMPs depends on the molecular balance between MMPs and TIMPs [39–41].

Integrins are cell surface receptors that meditate communication between cells and TME. They promote tumour angiogenesis, tumour cell proliferation and metastasis formation [42]. Integrins promote cancer growth in several ways. For example, VEGF can activate integrins leading to angiogenesis, or integrins can increase migration and invasion by activating proteases to remodel TME [43].

### (e) Extracellular vesicles

Despite the importance of soluble proteins in the modulation of the intercellular communication at primary and secondary tumour sites, EVs have emerged as key players in the crosstalk between cancer and TME cells [44,45]. EVs are a heterogeneous population of cell-derived vesicles enclosed by a lipid bilayer ranging in size from 30 to 2000 nm, and are classified based on their cellular origin, biological function or biogenesis [46,47]. EVs contain bioactive molecules, such as nucleic acids (DNA, mRNA, microRNA and other non-coding RNAs), proteins (receptors, transcription factors, enzymes and ECM proteins) and lipids, all of which can redirect the function of the recipient cell [48].

EVs preserve the bioactivity of their molecular cargo and can be readily isolated from multiple biological fluids (such as urine, serum, plasma, pleural effusion and saliva), suggesting that these particles may constitute potential biomarkers for the real-time assessment of cancer progression [49]. Differential ultracentrifugation stands as a conventional and the most widely used method to isolate different vesicles, but no consensus regarding a ‘gold standard’ method for the isolation or purification of EVs currently exists. The most efficient method depends primarily on the specific question asked in experiments as well as the downstream application of the particular EVs [50].

In order to elicit functional effects, EVs dock to target cells and initiate signalling events either at the cell surface or within cells. In either case, EVs are capable of promoting phenotypic changes in recipient cells depending on their cargo [51]. Specifically, in TME EVs mediate heterotypic interactions between stromal and cancer cells supporting fundamental cancer hallmarks, such as evading growth suppressors, resisting cell apoptosis, sustaining proliferative signalling, evading immune destruction and inducing migration, invasion and angiogenesis [52].

ECM remodelling is generally thought to promote invasive tumour phenotypes, including oral tongue cancer [53–55]. Secreted tumour EVs coated with fibronectin promote nascent adhesion assembly and increase cell motility [56]. Interestingly, we recently showed that a high concentration of fibronectin in oral tongue squamous cell carcinoma (OTSCC) TME associated with a poor prognosis, especially with early stage tumours, indicating a pro-carcinogenic role for TME fibronectin [57]. The mutual interplay between cancer cells and fibroblasts is thus crucial for cancer progression and EVs from cells actively participating in this process [58]. Furthermore, TGF-β associated with tumour EVs can trigger fibroblast differentiation into CAFs. Additionally, the ECM metalloproteinase inducer (EMMPRIN) found in the microvesicles shed by tumour cells can enhance the production of MMPs in fibroblasts, enabling tumour invasion and metastasis [59]. Similarly, recent work within our group demonstrated that EVs derived from CAFs can increase the invasiveness of tumours in oral squamous and colorectal carcinoma cell lines (MR Dourado 2016, unpublished data). By contrast, endothelial cells stimulated by VEGF and FGF-2 release EVs containing MMPs that initiate the proteolysis necessary for tumour invasion and uninhibited angiogenesis [60].

## 2. Three-dimensional *in vitro* invasion assays using myoma discs

### (a) Properties of myoma tissue discs

**Figure 1.**
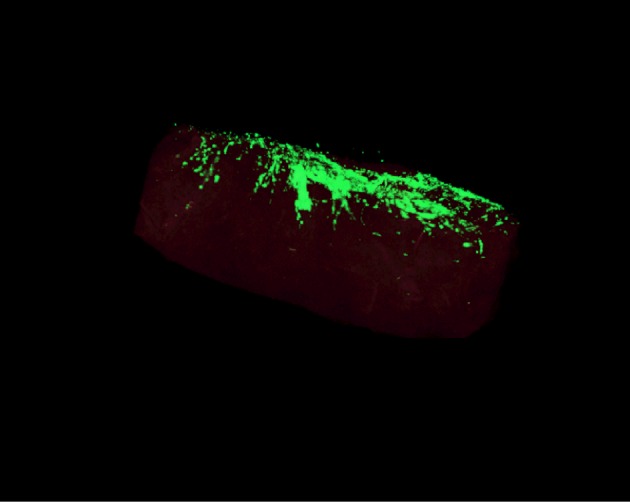
GFP-transfected HSC-3 squamous cell carcinoma cell line invading in 3D Myoma disc.

**Figure 2.**
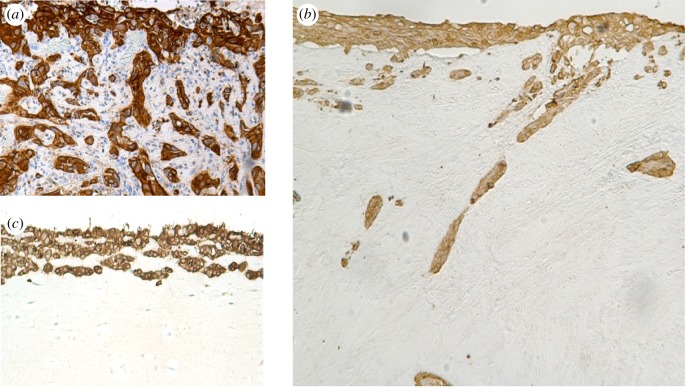
Oral tongue highly invasive HSC-3 squamous cell carcinoma cell line shows similar invading pattern to carcinoma cells *in vivo*. Pancytokeratin staining of patient section of an oral tongue squamous cell carcinoma (*a*), HSC-3 cell line in organotypic type I collagen and fibroblast disc section (*b*), and in Myoma disc (*c*).

**Figure 3.**
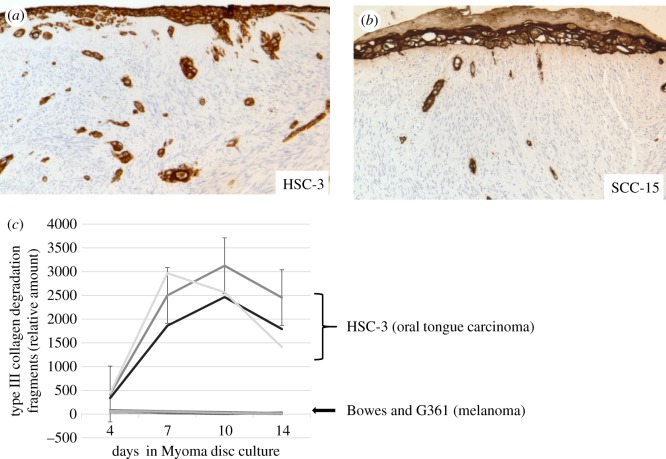
Cancer cells behave differently in Myoma disc. Highly invasive HSC-3 squamous cell carcinoma cell line invades deeply and covering a large invasion area (*a*), whereas less-invading SCC-15 shows less-invasive behaviour and forms a keratin layer (*b*). Degradation of type III collagen measured from the culture media of HSC-3 cell line (three separate experiments using myoma batches from three patients) and two different invasive melanoma cell lines (Bowes and G361; three separate experiments each), (*c*).

Ten years ago, we discovered that human uterus leiomyoma tumour–derived discs excellently mimic native TME for 3D cancer invasion assays ([61], figure 1). Unlike in myoma, no invasion of human oral tongue squamous cell carcinoma (HSC-3) cells was seen in a similar 3D set-up of discs prepared from a normal pig tongue or a human heart [55]. From an average-size tumour, more than 100 ready-to-use (diameter 8 mm, height 4 mm) discs can be prepared. In comparison to similarly sized 3D invasion discs made from rat tail type I collagen [62] with or without EHS mouse sarcoma-derivative Matrigel^®^, myoma discs provide a natural, complete human TME matrix. As an aggressive OTSCC cell line, HSC-3 cells penetrate seven times more profoundly in myoma than in collagen discs ([61], figure 2). Various solid cancer cell lines tested in myoma show that, in general, the more invasive the cells are *in vivo*, the deeper they invade in myoma discs (table 1). Instead, the less invasive OTSCC cells began to secrete keratin, unlike the more aggressive cell line (figure 3). Within myoma, the invasion patterns of OTSCC mimic the mesenchymal, collective or budding types seen in *in vivo* patient tissue sections. Some carcinoma cells within myoma discs demonstrate an epithelial-to-mesenchymal transition positive for both mesenchymal and epithelial cell markers [61]. In electron microscopy analyses, HSC-3 cells on top of myoma discs produce basement membrane (BM) structures, whereas deeper invading cells protrude invadopodia surrounded by broken BM fragments [55].

**Table 1.** Cell lines tested in myoma discs and Myogel using scratch wound assay. The invasiveness of various cell lines is marked using a scale from **–** (non-invading) to **+++** (highly invading). UD, unpublished data, LE, locally established primary cell lines.

cell line	tissue of origin	invasiveness in myoma disc	faster in Myogel versus Matrigel^®^ (unpublished data)		reference and additional data
			IncuCyte	Transwell	
HSC-3	tongue squamous cell carcinoma	+++*	Myogel	Myogel	[61]*
SCC-25	tongue squamous cell carcinoma	++*	Myogel	Myogel	[55]*
SCC-15	tongue squamous cell carcinoma	+*			[55]*
SAS	tongue squamous cell carcinoma	+++*		Myogel	[53]*
UT-SCC-43	tongue squamous cell carcinoma	+*			[63]*
DOK	dysplastic oral keratinocytes	+/−*			[61]*
ODA*	HPV 16 immortalized mucosal keratinocytes		Myogel		[64]*
HMK*	immortalized gingival keratinocytes	+**	Myogel		[65]*, UD**
PE/CA-PJ15	neoplastic oral keratinocytes	+*			UD*
UK-1	oro-pharyngeal keratinocytes	+*			[61]*
MDA-MB-231	mammary gland, breast	++*	Myogel = Matrigel^®^		[61]*
MDA-MB-435	melanoma	+*			UD*
Bowes	melanoma	+++*		Myogel	[61]*
G361	melanoma	++*			[61]*
HT1080	fibrosarcoma	+*		Matrigel^®^	UD*
HaCat	human keratinocytes	+/−*	Myogel		[66]*
UT-MUC-1	mucoepidermoid carcinoma	++*			[67]*
SK-MES-1	lung squamous cell carcinoma	++­+ *			[68]*
SK-LU-1	lung adenocarcinoma	+*			[68]*
HuH-7	hepatocellular carcinoma	+*			UD*
Mahlavu	hepatocellular carcinoma	++*			UD*
HCT-11	colon adenocarcinoma	++*			UD*
Colo-741	colon adenocarcinoma	+++*			UD*
BEAS-2B	non-malignant bronchial cells	++*			[68]*
MSCs	mesenchymal stem cells	+/−*			[69]*
JEG-3	choriocarcinoma cells	+*			[70]*
Pa01	liver ductal adenocarcinoma		Myogel		
Pa02c	liver ductal adenocarcinoma		Myogel	Myogel	
Pa03c	liver ductal adenocarcinoma		Myogel	Myogel	
Pa04c	liver ductal adenocarcinoma		no invasion	Myogel	
ASC*	adipocyte stem cells		Myogel = Matrigel^®^		*LE
IGF*	gingival fibroblasts		Myogel = Matrigel^®^		*LE
CAF*	oral carcinoma–associated fibroblasts		Myogel / Not tested in Matrigel^®^		*LE
MEK*	high-grade mucoepidermoid carcinoma	(**+**)**	No invasion		*LE **UD

Myoma discs comprise ECM structural proteins, such as type I, III and IV collagens and laminins [61]. TME cells, including fibroblasts, endothelial and smooth muscle cells, as well as CD68^+^ and CD45^+^ inflammatory cells, are also present [61]. Yet during the 3D culture procedure they all remain non-vital [71]. Depending on the myoma, the number of ECM molecules varies. After analysing myoma from more than 150 patients, we may conclude that the greater the amount of tenascin-C within the myoma discs, the deeper the cancer cells invade [55]. Interestingly, tenascin-C, especially in TME, serves as a marker for a poor prognosis in several solid cancers, including OTSCC [57]. As a result for this heterogeneity with the Myoma discs, all the experiments should be done using one single batch of Myoma to avoid such variations within the experiments. Similar variations are also known when using Matrigel and other animal-based ECM [72,73].

Native myoma discs are hypoxic [63]. Hypoxia in solid tumours induces invasion and affects, for instance, cell metabolism and angiogenesis [74]. When myoma discs are effectively rinsed, the soluble factors, including binding proteins, growth factor receptors and growth factors, are released. Some of these growth factors include HGF and fibroblast growth factor 2 (FGF2) and transforming growth factor beta (TGF-β) 1 and 2, which are known to affect cell motility [55]. Other soluble molecules in rinsing include MMP-11, lysyl oxidase-1 (LOX-1) and carbonic anhydrase IX (CA-9), all of which have been shown to facilitate cancer invasion *in vivo* [63]. Interestingly, intact myoma discs induced the upregulation of the urokinase plasminogen activator receptor (uPAR), which at the invasive tumour front is indicative of a poor prognosis in oral cancer [74]. In rinsed myoma, the invasion depth is significantly diminished compared with that in the corresponding native disc. However, in rinsed myoma, the type III collagen degradation, measured using a radioimmunoassay of a type III collagen fragment carboxy-terminal telopeptide in a myoma culture medium, increases. This suggests a greater need for the enzymatic degradation of collagen in order for cancer cells to invade cancer cells in rinsed rather than in native discs [63]. This shift in the invasion mechanism is also seen in lyophilized and rehydrated myoma discs. The invasion depth remains the same, although type III collagen degradation is higher in lyophilized discs compared with the corresponding native discs [55]. This system is suitable for measuring the invasion of cancer cells that degrade enzymatically myoma collagen, such as OTSCC cell lines. In the case of melanoma cells, which use non-enzymatic invasion mechanism in myoma, the radioimmunoassay method is not suitable (figure 3*c*).

Myoma discs are used to compare the invasion of parental and transduced cell lines, such as MMP-8, endostatin, urokinase plasminogen activator receptor (uPAR) and trypsin-2 overexpressed, or cathepsin K, snail, miRNA-498 and miRNA-940 silenced carcinoma cell lines [39,54,68,71,75–77]. Similarly, several compounds have been studied for their effects on invasion. Specifically, we investigated aptamers against heparanase, TLR antagonists, TLR ligands and MMP inhibitor GM6001 [61,67,78–80]. We also used myoma disc invasion assays to test monoclonal antibodies against chemokine CCL5, and the drug β-aminoproprionitrile, which inhibits lysyl oxidase [53,63].

### (b) Cocultures for carcinoma and cancer-associated fibroblasts or inflammatory cells in myoma discs

It is quite obvious that coculturing mesenchymal cells, such as CAFs, and immune cells together with cancer cells in a two-dimensional (2D) setting is far from truly simulating the real interaction between these cells. This is due to several factors, such as the omission of native ECM, different adherence behaviour between cells and the loss of circulating immune cells.

In order to develop coculturing techniques, the Transwell technique was developed to provide a barrier between different cell lines. Transwell was first introduced in 1989 by Repesh, and primarily used initially to study cancer cell invasion [81]. Later, Transwell was applied to coculture cancer cells with other types of cells, including fibroblasts and immune cells. With the introduction of Matrigel^®^ and other synthesised matrices, researchers began to shift from 2D towards 3D cell cultures. To some extent, these matrices provided ECM to cells, but the problem of the missing human ECM–based matrix with tumour features remained unsolved.

In order to overcome the lack of human-based ECM, we developed a coculture system using human myoma tumour discs ([61], see above). This system was first used to coculture OTSCC with bone marrow mesenchymal stem cells (BMMSC) [53], fibroblasts [82] and macrophages [83]. Adding OTSCC together with either BMMSC or tumour-related macrophages (M2) stimulated from THP-1 cells induced carcinoma cell invasion, unlike normal fibroblast and inflammation-associated M1 macrophages, which reduced invasion when compared with OTSCC cultures [53,83].

Subsequently, we further developed the system to coculture OTSCC and non-adherent immune cells. The system is based on culturing cancer cells on top of the myoma discs and adding the immune cells (with or without stimulants) to the bottom of the discs. This system enable us not only to study the effect of the immune cells on cancer cells, but also to study the recruitment of the immune cells towards cancer cells [84]. This system is efficient, but also faces the problem of missing circulation for the immune cells.

## 3. Myogel preparation and properties

The human uterus leiomyoma tumour–derived matrix Myogel is prepared using a method similar to that for the production of Engelbreth–Holm–Swarm (EHS) mouse sarcoma–derived commercial products, such as Matrigel^®^, ECMatrix™, Cultrex^®^, BME^®^ or Geltrex^®^. Myoma tissue is ground to a powder, suspended in a sodium chloride buffer and, after centrifugation, the pellet is homogenized in the same buffer followed by protein concentration measurement. Only about one-third of proteins in Myogel are similar to those in Matrigel^®^. The same proteins in both include, for example, laminin, type IV collagen, nidogen, heparan sulfate proteoglycans and epidermal growth factor. Unlike Myogel, Matrigel^®^ contains entactin, whereas tenascin-C, an important component in several tumour matrices, is present only in Myogel [55,85]. Myogel has both latent and active forms of MMP-2, whereas Matrigel^®^ contains both gelatinases MMP-2 and MMP-9.

Commercial products prepared from either human tissue or cocultured human cells, such as skeletal muscle Myogel [86], amnion tissue extract [87] and a fibroblast–keratinocyte coculture matrix are available, but these all mimic normal ECM. None are derived from tumour tissue ECM. In cell culture conditions, the pH of Myogel remains neutral and stable compared with Matrigel^®^ which is more acidic. OTSCC cells (HSC-3) adhere better on top of BM-mimicking Matrigel^®^ compared with the top of TME-mimicking Myogel; however, the migration of those cells on top of Myogel is faster compared with the top of Matrigel^®^ [85]. Compared with plastic, HSC-3 cells on top of Myogel changed the expression of 1.4% of the genes, specifically those related to intracellular organelle, cytoskeleton organisation and biogenesis [85].

Myogel, a human TME matrix, is thus ideal for the study of the human cancer cell expression profile and movement in 3D *in vitro* conditions.

In the following sections, we describe Myogel use in various experiments, such as Transwell invasion, scratch assay, spheroid (hanging-drop and round-bottom ULA-plate assay) and sandwich assays, as well as tube formation assays. Additionally, Myogel has been used in high-throughput cancer drug testing.

### (a) Cancer invasion assays using Myogel

*In vitro* invasion assays quantify cells invading through structures such as basement membrane equivalents, reconstituted collagen gels or more complex materials [88]. By coating the porous filter with ECM components, the Transwell system suitably tests invasion. In both cases, either the invaded or migrated cells are assessed by counting the cells that penetrate the insert, either through visual counting after staining with dye or fluorescence after lysing invading or migrating fluorescently labelled cells [5,89]. We showed that several cancer cell lines invade more efficiently through Transwell coated with Myogel than when coated with Matrigel^®^ [85] (table 1). The same system can also be used to study EVs in cancer migration and invasion, in direct contact with cancer cells and using a coculture method.

The IncuCyte^®^ system is a platform that allows real-time imaging of vertical and horizontal cell migration and invasion, using either label-free or dual-colour fluorescence to study specific cell populations in coculture. The traditional scratch wound healing assay can be performed using a special Image Lock 96-well plate using the WoundMaker™ on a confluent cell monolayer creating a uniform wound area along the wells, allowing measurement of cell movement into the wound. Coating of the plates with, for instance, collagen I, collagen IV, fibronectin, Matrigel^®^ or Myogel prior to cell seeding allows us to study migration on different substrates [89]. In doing so, the differential biology of cell migration and invasion can be explored within the same plate. After creating the wound and washing to remove dead cells and debris, a biomatrix can be added on top of the wounded cell monolayer, and the horizontal cell invasion into the wound can be monitored. When working with EVs or for drugs testing, the compound/vesicles can be added straight into the medium for migration and invasion or into the biomatrix in the invasion assay. Results are then calculated based on the speed of wound closure. Using zooming tools, we can monitor the morphological changes of the cells during the movement process. This platform has a special 96-well plate resembling the Transwell system to assess chemotactic migration and invasion. Based on a similar idea, the well has two chambers separated by a porous membrane. The imaging system allows for the real-time monitoring of vertically invading (through a biomatrix and membrane) and migrating cells.

**Figure 4.**
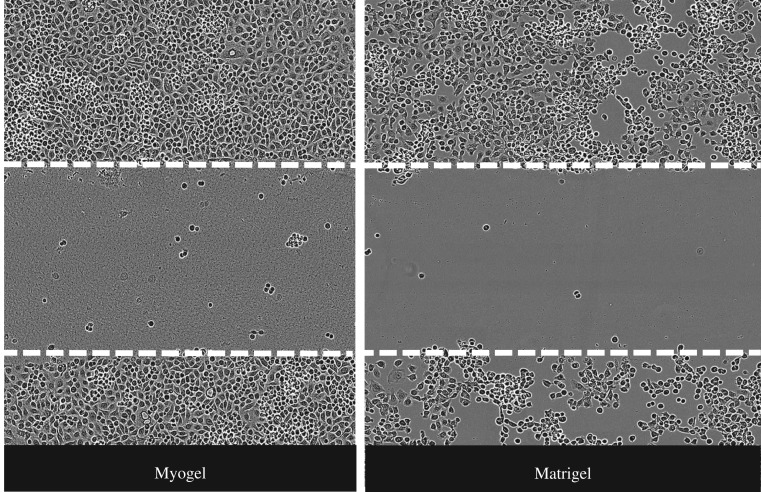
HSC-3 cells are invading faster in Myogel than in Matrigel^®^ in IncuCyte Zoom system (video).

**Figure 5.**
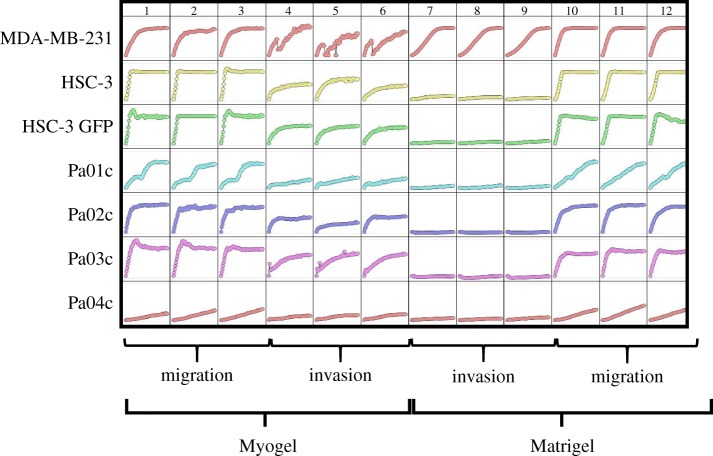
Cancer cells invade differently in Myogel compared with Matrigel^®^. Migration and invasion of different cancer cell types were tested in Myogel and Matrigel^®^ using IncuCyte Zoom system. (See the cell lines table 1.)

Similar to the Transwell assay, several cancer cell lines invade faster within Myogel than within Matrigel^®^ in the IncuCyte^®^ system using scratch wound assay (figures 4 and 5 and table 1).

### (b) Spheroid and sandwich assays using Myogel

Cells can create aggregates—spheroids—which are used for measuring molecular or biomechanical properties. In hanging-drop spheroids, small droplets of cells are first embedded within various matrices, such as type I collagen, or a mixture of Matrigel^®^ and type I collagen or Myogel and type I collagen. Cell suspension in matrix drops is placed at the centre of each compartment, and, after a short incubation, the plate is inverted. A confocal fluorescence microscope allows for time-lapse imaging. For fluorescent detection, the cells are either transfected with lentiviral particles (nuclear or cytoplasmic) or stained using a lipophilic-membrane stain that diffuses laterally. Different cell invasion parameters, such as the speed, volume and cell shape, are analysed using image analysis software. We have shown that the invasion speed of HSC-3 cells was fastest in a Myogel–collagen compared with only collagen or Matrigel^®^–collagen matrices in this hanging-drop assay. In a round-bottom well ULA plate assay [90], analysing MRC-5 fibroblasts, pancreas cancer (Patu 8988T) cells and their cocultures both fibroblasts and cancer cells grew significantly larger in Myogel than in Matrigel^®^. However, the sizes of the cocultures grown in either matrix remained the same (R Chopra, 2016, unpublished data).

In a sandwich assay, fluorescent-labelled cancer cells are embedded between two layers of polymerised gel matrices. The 3D ECM sandwich assay is ideal for long-term imaging. This method can be performed in 96-well angiogenesis plates consisting of two compartments: one with a smaller bottom and a larger upper one. The bottom section is filled with an ECM mixture, and after polymerization an ECM mixture embedded with cells is added to the upper compartment. In the upper compartment, a culture medium is added prior to confocal fluorescence microscopy. This method is ideal for visualization involving cells all located on one level, unlike the case for hanging-drop aggregates. Our preliminary data show that HSC-3 cells feature smaller nuclei and move faster in Myogel–collagen (EH Apu 2015, unpublished data) than in a Matrigel^®^ collagen, and breast cancer cells (MDA-MB-231) formed invasive properties in a Myogel collagen (M Nees 2016, unpublished data).

### (c) Tube formation assay using Myogel

In Myogel, a greater number of endothelial cells formed along with smaller tubes compared with Matrigel^®^, which is typically used for angiogenesis experiments. In both matrices, the cellular network structures were fully developed by 12 h, but tube formation in Myogel continued for as long as 72 h; at that time point in Matrigel^®^ the endothelial cells were already in apoptosis [85]. Therefore, vessel formation assay in Myogel could be used for pro- or anti-angiogenic drug assays where the number of tubes represents the most crucial factor being evaluated.

### (d) Myogel in three-dimensional cancer drug testing

For cancer drug testing, a quantitative drug sensitivity score, based on continuous modelling and integration of multiple dose–response relationships in a high-throughput compound test, was created [32]. Using this method, we tested 135 different drugs against pancreas carcinoma (Pa02c) cells in 384 well plates, which were either uncoated or pre-coated with Matrigel^®^ or Myogel. Surprisingly, roughly 20% of the drugs yielded different results in the viability test (ATP amount) when cells were cultured on coated versus uncoated plates (K Tuomainen 2016, unpublished data). These preliminary results suggest that cancer drugs should be tested on cells cultured in contact with ECM rather than simply on top of plastic.

## 4. Conclusion

Myoma tissue products, created from leftover tumour pieces following human uterus myoma removal, appear ethically superior to sacrificing thousands of mice with basement membrane–producing EHS tumours used to produce Matrigel^®^. Myoma discs and Myogel, the only available human tumour-derived ECM products, appear completely suitable for numerous solid cancer-related *in vitro* assays (migration, invasion, radiation or drug testing). In future, we wish that myoma discs and Myogel will ideally replace animal tissue–derived matrices, such as Matrigel^®^ and type I collagen, for 3D experiments. In theory, Myogel and myoma discs may well be used in future for ‘personalized medicine’ cancer assays.

## Data accessibility

This article has no additional data.

## References

[1] Erdogan B, Webb DJ 2017 Cancer-associated fibroblasts modulate growth factor signaling and extracellular matrix remodeling to regulate tumor metastasis. Biochem. Soc. Trans. 45, 229–236. (10.1042/BST20160387) 28202677 PMC5371349

[2] Kam Y, Rejniak KA, Anderson AR 2012 Cellular modeling of cancer invasion: integration of *in silico* and *in vitro* approaches. J. Cell Physiol. 227, 431–438. (10.1002/jcp.22766) 21465465 PMC3687536

[3] Hanahan D, Weinberg RA 2011 Hallmarks of cancer: the next generation. Cell 144, 646–674. (10.1016/j.cell.2011.02.013) 21376230

[4] Pandya P, Orgaz JL, Sanz-Moreno V 2017 Modes of invasion during tumour dissemination. Mol. Oncol. 11, 5–27. (10.1002/1878-0261.12019) 28085224 PMC5423224

[5] Menyhárt O, Harami-Papp H, Sukumar S, Schäfer R, Magnani L, de Barrios O, Győrffy B 2016 Guidelines for the selection of functional assays to evaluate the hallmarks of cancer. Biochim. Biophys. Acta 1866, 300–319. (10.1016/j.bbcan.2016.10.002) 27742530

[6] Direkze NC, Hodivala-Dilke K, Jeffery R, Hunt T, Poulsom R, Oukrif D, Alison MR, Wright NA 2004 Bone marrow contribution to tumor-associated myofibroblasts and fibroblasts. Cancer Res. 64, 8492–8495. (10.1158/0008-5472.CAN-04-1708) 15574751

[7] Potenta S, Zeisberg E, Kalluri R 2008 The role of endothelial-to-mesenchymal transition in cancer progression. Br. J. Cancer 99, 1375–1379. (10.1038/sj.bjc.6604662) 18797460 PMC2579683

[8] Järvinen PM, Laiho M 2012 LIM-domain proteins in transforming growth factor β-induced epithelial-to-mesenchymal transition and myofibroblast differentiation. Cell. Signal. 24, 819–825. (10.1016/j.cellsig.2011.12.004) 22182513

[9] Rønnov-Jessen L, Petersen OW, Koteliansky VE, Bissell MJ 1995 The origin of the myofibroblasts in breast cancer. Recapitulation of tumor environment in culture unravels diversity and implicates converted fibroblasts and recruited smooth muscle cells. J. Clin. Invest. 95, 859–873. (10.1172/JCI117736) 7532191 PMC295570

[10] Gaggioli C, Hooper S, Hidalgo-Carcedo C, Grosse R, Marshall JF, Harrington K, Sahai E 2007 Fibroblast-led collective invasion of carcinoma cells with differing roles for RhoGTPases in leading and following cells. Nat. Cell Biol. 9, 1392–1400. (10.1038/ncb1658) 18037882

[11] Cai J, Tang H, Xu L, Wang X, Yang C, Ruan S, Guo J, Hu S, Wang Z 2012 Fibroblasts in omentum activated by tumor cells promote ovarian cancer growth, adhesion and invasiveness. Carcinogenesis 33, 20–29. (10.1093/carcin/bgr230) 22021907

[12] Vered M, Dayan D, Yahalom R, Dobriyan A, Barshack I, Bello IO, Kantola S, Salo T 2010 Cancer-associated fibroblasts and epithelial-mesenchymal transition in metastatic oral tongue squamous cell carcinoma. Int. J. Cancer 127, 1356–1362. (10.1002/ijc.25358) 20340130

[13] Bello IO et al. 2011 Cancer-associated fibroblasts, a parameter of the tumor microenvironment, overcomes carcinoma-associated parameters in the prognosis of patients with mobile tongue cancer. Oral Oncol. 47, 33–38. (10.1016/j.oraloncology.2010.10.013) 21112238

[14] Özdemir BC et al. 2014 Depletion of carcinoma-associated fibroblasts and fibrosis induces immunosuppression and accelerates pancreas cancer with reduced survival. Cancer Cell 25, 719–734. (10.1016/j.ccr.2014.04.005) 24856586 PMC4180632

[15] Ostman A, Augsten M 2009 Cancer-associated fibroblasts and tumor growth--bystanders turning into key players. Curr. Opin. Genet. Dev. 19, 67–73. (10.1016/j.gde.2009.01.003) 19211240

[16] Orr B, Riddick AC, Stewart GD, Anderson RA, Franco OE, Hayward SW, Thomson AA 2012 Identification of stromally expressed molecules in the prostate by tag-profiling of cancer-associated fibroblasts, normal fibroblasts and fetal prostate. Oncogene 31, 1130–1142. (10.1038/onc.2011.312) 21804603 PMC3307063

[17] Kalluri R, Zeisberg M 2006 Fibroblasts in cancer. Nat. Rev. Cancer 6, 392–401. (10.1038/nrc1877) 16572188

[18] Shrihari TG 2017 Dual role of inflammatory mediators in cancer. Ecancermedicalscience 11, 721 (10.3332/ecancer.2017.721) 28275390 PMC5336391

[19] DeNardo DG, Coussens LM 2007 Inflammation and breast cancer. Balancing immune response: crosstalk between adaptive and innate immune cells during breast cancer progression. Breast Cancer Res. 9, 212 (10.1186/bcr1746) 17705880 PMC2206719

[20] Mantovani A, Sica A 2010 Macrophages, innate immunity and cancer: balance, tolerance, and diversity. Curr. Opin. Immunol. 22, 231–237. (10.1016/j.coi.2010.01.009) 20144856

[21] Sica A, Bronte V 2007 Altered macrophage differentiation and immune dysfunction in tumor development. J. Clin. Invest. 117, 1155–1166. (10.1172/JCI31422) 17476345 PMC1857267

[22] Laghi L et al. 2009 CD3+ cells at the invasive margin of deeply invading (pT3-T4) colorectal cancer and risk of post-surgical metastasis: a longitudinal study. Lancet. Oncol. 10, 877–884. (10.1016/S1470-2045(09)70186-X) 19656725

[23] Pages F et al. 2005 Effector memory T cells, early metastasis, and survival in colorectal cancer. N. Engl. J. Med. 353, 2654–2666. (10.1056/NEJMoa051424) 16371631

[24] Dayan D, Salo T, Salo S, Nyberg P, Nurmenniemi S, Costea DE, Vered M 2012 Molecular crosstalk between cancer cells and tumor microenvironment components suggests potential targets for new therapeutic approaches in mobile tongue cancer. Cancer Med. 1, 128–140. (10.1002/cam4.24) 23342263 PMC3544451

[25] Ostrand-Rosenberg S 2008 Immune surveillance: a balance between protumor and antitumor immunity. Curr. Opin. Genet. Dev. 18, 11–18. (10.1016/j.gde.2007.12.007) 18308558 PMC2699403

[26] Farkona S, Diamandis EP, Blasutig IM 2016 Cancer immunotherapy: the beginning of the end of cancer? BMC Med. 14, 73 (10.1186/s12916-016-0623-5) 27151159 PMC4858828

[27] Ascierto PA et al. 2016 Future perspectives in melanoma research: Meeting report from the ‘Melanoma Bridge’. Napoli, December 1st-4th 2015. J. Transl. Med. 14, 313 (10.1186/s12967-016-1070-y) 27846884 PMC5111349

[28] Ribas A et al. 2016 Association of pembrolizumab with tumor response and survival among patients with advanced melanoma. JAMA 315, 1600–1609. (10.1001/jama.2016.4059) 27092830

[29] Yin Q et al. 2017 Associations between tumor vascularity, vascular endothelial growth factor expression and PET/MRI radiomic signatures in primary clear-cell-renal-cell-carcinoma: proof-of-concept study. Sci. Rep. 7, 43356 (10.1038/srep43356) 28256615 PMC5335708

[30] Nyberg P, Salo T, Kalluri R 2008 Tumor microenvironment and angiogenesis. Front. Biosci. 13, 6537–6553. (10.2741/3173) 18508679

[31] Karamysheva AF 2008 Mechanisms of angiogenesis. Biochemistry 73, 751–762. 18707583 10.1134/s0006297908070031

[32] Yadav B et al. 2014 Quantitative scoring of differential drug sensitivity for individually optimized anticancer therapies. Sci. Rep. 4, 5193 (10.1038/srep05193) 24898935 PMC4046135

[33] Morikawa S, Baluk P, Kaidoh T, Haskell A, Jain RK, McDonald DM 2002 Abnormalities in pericytes on blood vessels and endothelial sprouts in tumors. Am. J. Pathol. 160, 985–1000. (10.1016/S0002-9440(10)64920-6) 11891196 PMC1867175

[34] Hida K, Maishi N, Torii C, Hida Y 2016 Tumor angiogenesis--characteristics of tumor endothelial cells. Int. J. Clin. Oncol. 21, 206–212. (10.1007/s10147-016-0957-1) 26879652

[35] Harris AL 2002 Hypoxia--a key regulatory factor in tumour growth. Nat. Rev. Cancer 2, 38–47. (10.1038/nrc704) 11902584

[36] Gkretsi V, Stylianou A, Papageorgis P, Polydorou C, Stylianopoulos T 2015 Remodeling components of the tumor microenvironment to enhance cancer therapy. Front. Oncol. 5, 214 (10.3389/fonc.2015.00214) 26528429 PMC4604307

[37] Klein G, Vellenga E, Fraaije MW, Kamps WA, de Bont ES 2004 The possible role of matrix metalloproteinase (MMP)-2 and MMP-9 in cancer, e.g. acute leukemia. Crit. Rev. Oncol. Hematol. 50, 87–100. (10.1016/j.critrevonc.2003.09.001) 15157658

[38] Wang K, Wu F, Seo BR, Fischbach C, Chen W, Hsu L, Gourdon D 2017 Breast cancer cells alter the dynamics of stromal fibronectin-collagen interactions. Matrix. Biol. 60–61, 86–95. (10.1016/j.matbio.2016.08.001) PMC5293676 27503584

[39] Åström P 2014 Regulatory mechanisms mediating matrix metalloproteinase-8 effects in oral tissue repair and tongue cancer. Acta Universitatis Ouluensis D Medica 1263. Juvenes print, Tampere-Finland.

[40] Cauwe B, Van den Steen PE, Opdenakker G 2007 The biochemical, biological, and pathological kaleidoscope of cell surface substrates processed by matrix metalloproteinases. Crit. Rev. Biochem. Mol. Biol. 42, 113–185. (10.1080/10409230701340019) 17562450

[41] Arpino V, Brock M, Gill SE 2015 The role of TIMPs in regulation of extracellular matrix proteolysis. Matrix. Biol. 44–46, 247–254. (10.1016/j.matbio.2015.03.005) 25805621

[42] Alphonso A, Alahari SK 2009 Stromal cells and integrins: conforming to the needs of the tumor microenvironment. Neoplasia 11, 1264–1271. (10.1593/neo.91302) 20019834 PMC2794507

[43] White DE, Muller WJ 2007 Multifaceted roles of integrins in breast cancer metastasis. J. Mammary Gland Biol. Neoplasia 12, 135–142. (10.1007/s10911-007-9045-5) 17602286

[44] Costa-Silva B et al. 2015 Pancreatic cancer exosomes initiate pre-metastatic niche formation in the liver. Nat. Cell Biol. 17, 816–826. (10.1038/ncb3169) 25985394 PMC5769922

[45] Mathias RA, Gopal SK, Simpson RJ 2013 Contribution of cells undergoing epithelial-mesenchymal transition to the tumour microenvironment. J. Proteomics 78, 545–557. (10.1016/j.jprot.2012.10.016) 23099347

[46] Gudbergsson JM, Johnsen KB, Skov MN, Duroux M 2016 Systematic review of factors influencing extracellular vesicle yield from cell cultures. Cytotechnology 68, 579–592. (10.1007/s10616-015-9913-6) 26433593 PMC4960200

[47] El Andaloussi S, Lakhal S, Mäger I, Wood MJ 2013 Exosomes for targeted siRNA delivery across biological barriers. Adv. Drug. Deliv. Rev. 65, 391–397. (10.1016/j.addr.2012.08.008) 22921840

[48] Raposo G, Stoorvogel W 2013 Extracellular vesicles: exosomes, microvesicles, and friends. J. Cell Biol. 200, 373–383. (10.1083/jcb.201211138) 23420871 PMC3575529

[49] Weis SM, Cheresh DA 2011 Tumor angiogenesis: molecular pathways and therapeutic targets. Nat. Med. 17, 1359–1370. (10.1038/nm.2537) 22064426

[50] Lötvall J *et al* 2014 Minimal experimental requirements for definition of extracellular vesicles and their functions: a position statement from the international society for extracellular vesicles. J. Extracell. Vesicles 3, 26913 (10.3402/jev.v3.26913) 25536934 PMC4275645

[51] French KC, Antonyak MA, Cerione RA 2017 Extracellular vesicle docking at the cellular port: extracellular vesicle binding and uptake. Semin. Cell Dev. Biol. 67, 48–55. (10.1016/j.semcdb.2017.01.002) 28104520 PMC5484727

[52] Gopal SK, Greening DW, Rai A, Chen M, Xu R, Shafiq A, Mathias RA, Zhu HJ, Simpson RJ 2017 Extracellular vesicles: their role in cancer biology and epithelial-mesenchymal transition. Biochem. J. 474, 21–45. (10.1042/BCJ20160006) 28008089

[53] Salo S et al. 2013 Human bone marrow mesenchymal stem cells induce collagen production and tongue cancer invasion. PLoS ONE 8, e77692. (doi:10.1371/journal.pone.0077692. Erratum in: *PLoS ONE* 2013; **8**(11)) 24204919 10.1371/journal.pone.0077692 PMC3804615

[54] Bitu CC, Kauppila JH, Bufalino A, Nurmenniemi S, Teppo S, Keinänen M, Vilen ST, Lehenkari P 2013 Cathepsin K is present in invasive oral tongue squamous cell carcinoma in vivo and in vitro. PLoS ONE 8, e70925 (10.1371/journal.pone.0070925) 23951042 PMC3737264

[55] Sundquist E et al. 2016 Neoplastic extracellular matrix environment promotes cancer invasion in vitro. Exp. Cell Res. 344, 229–240. (10.1016/j.yexcr.2016.04.003) 27090016

[56] Sung BH, Ketova T, Hoshino D, Zijlstra A, Weaver AM 2015 Directional cell movement through tissues is controlled by exosome secretion. Nat. Commun. 6, 7164 (10.1038/ncomms8164) 25968605 PMC4435734

[57] Sundquist E et al. 2017 Tenascin-C and fibronectin expression divide early stage tongue cancer into low- and high-risk groups. Br. J. Cancer 116, 640–648. (10.1038/bjc.2016.455) 28095396 PMC5344290

[58] Ciardiello C, Cavallini L, Spinelli C, Yang J, Reis-Sobreiro M, de Candia P, Minciacchi VR, Di Vizio D 2016 Focus on extracellular vesicles: new frontiers of cell-to-cell communication in cancer. Int. J. Mol. Sci. 17, 175 (10.3390/ijms17020175) 26861306 PMC4783909

[59] Sidhu SS, Mengistab AT, Tauscher AN, LaVail J, Basbaum C 2004 The microvesicle as a vehicle for EMMPRIN in tumor-stromal interactions. Oncogene 23, 956–963. (10.1038/sj.onc.1207070) 14749763

[60] Taraboletti G, D'Ascenzo S, Borsotti P, Giavazzi R, Pavan A, Dolo V 2002 Shedding of the matrix metalloproteinases MMP-2, MMP-9, and MT1-MMP as membrane vesicle-associated components by endothelial cells. Am. J. Pathol. 160, 673–680. (10.1016/S0002-9440(10)64887-0) 11839588 PMC1850663

[61] Nurmenniemi S, Sinikumpu T, Alahuhta I, Salo S, Sutinen M, Santala M, Risteli J, Nyberg P, Salo T 2009 A novel organotypic model mimics the tumor microenvironment. Am. J. Pathol. 175, 1281–1291. (10.2353/ajpath.2009.081110) 19679876 PMC2731146

[62] Fusenig NE, Amer SM, Boukamp P, Worst PK 1978 Characteristics of chemically transformed mouse epidermal cells in vitro and in vivo. Bull. Cancer 65, 271–279. 102384

[63] Teppo S et al. 2013 The hypoxic tumor microenvironment regulates invasion of aggressive oral carcinoma cells. Exp. Cell Res. 319, 376–389. (10.1016/j.yexcr.2012.12.010) 23262025

[64] Oda D, Bigler L, Lee P, Blanton R 1996 HPV immortalization of human oral epithelial cells: a model for carcinogenesis. Exp. Cell Res. 226, 164–169. (10.1006/excr.1996.0215) 8660952

[65] Mäkelä M, Salo T, Larjava H 1998 MMP-9 from TNF alpha-stimulated keratinocytes binds to cell membranes and type I collagen: a cause for extended matrix degradation in inflammation? Biochem. Biophys. Res. Commun. 253, 325–335. (10.1006/bbrc.1998.9641) 9878537

[66] Omar AA et al. 2015 Toll-like receptors -4 and -5 in oral and cutaneous squamous cell carcinomas. J. Oral Pathol. Med. 44, 258–265. (10.1111/jop.12233) 25047824

[67] Korvala J et al. 2014 Toll-like receptor 9 expression in mucoepidermoid salivary gland carcinoma may associate with good prognosis. J. Oral Pathol. Med. 43, 530–537. (10.1111/jop.12160) 24484266

[68] Merikallio H, Turpeenniemi-Hujanen T, Pääkkö P, Mäkitaro R, Salo S, Salo T, Harju T, Soini Y 2012 Snail promotes an invasive phenotype in lung carcinoma. Respir. Res. 13, 104 (10.1186/1465-9921-13-104) 23157169 PMC3546026

[69] Nurmenniemi S et al. 2010 Toll-like receptor 9 ligands enhance mesenchymal stem cell invasion and expression of matrix metalloprotease-13. Exp. Cell Res. 316, 2676–2682. (10.1016/j.yexcr.2010.05.024) 20553713

[70] Lee CL, Chiu PC, Hautala L, Salo T, Yeung WS, Stenman UH, Koistinen H 2013 Human chorionic gonadotropin and its free β-subunit stimulate trophoblast invasion independent of LH/hCG receptor. Mol. Cell Endocrinol. 375, 43–52. (10.1016/j.mce.2013.05.009) 23684886

[71] Alahuhta I et al. 2015 Endostatin induces proliferation of oral carcinoma cells but its effect on invasion is modified by the tumor microenvironment. Exp. Cell Res. 336, 130–140. (10.1016/j.yexcr.2015.06.012) 26112215

[72] Hughes CS, Postovit LM, Lajoie GA 2010 Matrigel: a complex protein mixture required for optimal growth of cell culture. Proteomics 10, 1886–1890. (10.1002/pmic.200900758) 20162561

[73] Sharma NS, Nagrath D, Yarmush ML 2010 Adipocyte-derived basement membrane extract with biological activity: applications in hepatocyte functional augmentation in vitro. FASEB J. 24, 2364–2374. (10.1096/fj.09-135095) 20233948 PMC2887257

[74] Lu X, Kang Y 2010 Hypoxia and hypoxia-inducible factors: master regulators of metastasis. Clin. Cancer Res. 16, 5928–5935. (10.1158/1078-0432.CCR-10-1360) 20962028 PMC3005023

[75] Magnussen S et al. 2014 Tumour microenvironments induce expression of urokinase plasminogen activator receptor (uPAR) and concomitant activation of gelatinolytic enzymes. PLoS ONE 9, e105929 (10.1371/journal.pone.0105929) 25157856 PMC4144900

[76] Vilen ST et al. 2012 Trypsin-2 enhances carcinoma invasion by processing tight junctions and activating ProMT1-MMP. Cancer Invest. 30, 583–592. (10.3109/07357907.2012.716467) 22909050

[77] Korvala J et al. 2017 MicroRNA and protein profiles in invasive versus non-invasive oral tongue squamous cell carcinoma cells in vitro. Exp. Cell Res. 350, 9–18. (10.1016/j.yexcr.2016.10.015) 27773646

[78] Simmons SC et al 2014 Anti-heparanase aptamers as potential diagnostic and therapeutic agents for oral cancer. PLoS ONE 9, e96846 (10.1371/journal.pone.0096846) 25295847 PMC4189786

[79] Mäkinen LK, Ahmed A, Hagström J, Lehtonen S, Mäkitie AA, Salo T, Haglund C, Atula T 2016 Toll-like receptors 2, 4, and 9 in primary, metastasized, and recurrent oral tongue squamous cell carcinomas. J. Oral Pathol. Med. 45, 338–345. (10.1111/jop.12373) 26426362

[80] Kauppila JH et al. 2015 Toll-like receptor 9 mediates invasion and predicts prognosis in squamous cell carcinoma of the mobile tongue. J. Oral Pathol. Med. 44, 571–577. (10.1111/jop.12272) 25338738

[81] Repesh LA 1989 A new in vitro assay for quantitating tumor cell invasion. Invasion Metastasis 9, 192–208. 2722434

[82] Vered M et al. 2015 Caveolin-1 accumulation in the tongue cancer tumor microenvironment is significantly associated with poor prognosis: an in-vivo and in-vitro study. BMC Cancer 15, 25 (10.1186/s12885-015-1030-6) 25633184 PMC4318139

[83] Pirilä E et al. 2015 Macrophages modulate migration and invasion of human tongue squamous cell carcinoma. PLoS ONE 10, e0120895 (10.1371/journal.pone.0120895) 25811194 PMC4374792

[84] Al-Samadi A, Awad SA, Tuomainen K, Zhao Y, Salem A, Parikka M, Salo T 2017 Crosstalk between tongue carcinoma cells, extracellular vesicles, and immune cells in in vitro and in vivo models. Oncotarget 8, 60 123–60 134. 10.18632/oncotarget.17768 PMC5601126 28947958

[85] Salo T et al. 2015 A novel human leiomyoma tissue derived matrix for cell culture studies. BMC Cancer 15, 981 (10.1186/s12885-015-1944-z) 26673244 PMC4682271

[86] Abberton KM, Bortolotto SK, Woods AA, Findlay M, Morrison WA, Thompson EW, Messina A 2008 Myogel, a novel, basement membrane-rich, extracellular matrix derived from skeletal muscle, is highly adipogenic in vivo and in vitro. Cells Tissues Organs 188, 347–358. (10.1159/000121575) 18354248

[87] Yuan K, Kucik D, Singh RK, Listinsky CM, Listinsky JJ, Siegal GP 2008 Alterations in human breast cancer adhesion-motility in response to changes in cell surface glycoproteins displaying alpha-L-fucose moieties. Int. J. Oncol. 32, 797–807. 18360707 PMC2671470

[88] Inglehart RC, Scanlon CS, D'Silva NJ 2014 Reviewing and reconsidering invasion assays in head and neck cancer. Oral Oncol. 50, 1137–1143. (10.1016/j.oraloncology.2014.09.010) 25448226 PMC4254578

[89] Kramer N, Walzl A, Unger C, Rosner M, Krupitza G, Hengstschläger M, Dolznig H 2013 In vitro cell migration and invasion assays. Mutat. Res. 752, 10–24. (10.1016/j.mrrev.2012.08.001) 22940039

[90] Vinci M, Box C, Eccles SA 2015 Three-dimensional (3D) tumor spheroid invasion assay. J. Vis. Exp. 1, e52686 (10.3791/52686) PMC4542056 25993495

